# *In silico* Homology Modeling and Epitope Prediction of NadA as a Potential Vaccine Candidate in *Neisseria meningitidis*

**DOI:** 10.22088/IJMCM.BUMS.7.1.53

**Published:** 2018-02-10

**Authors:** Narjes Shahsavani, Mohammad Hasan Sheikhha, Hassan Yousefi, Fatemeh Sefid

**Affiliations:** 1 *Shahid Sadoughi University of Medical Sciences and Health Services, Yazd, Iran.*; 2 *Department of Medical Genetics, School of Medicine, Tehran University of Medical Sciences, Tehran, Iran.*; 3 *Department of Biology, Science and Arts University, Yazd, Iran.*

**Keywords:** Neisseria meningitidis, NadA, epitope prediction, 3D structure

## Abstract

*Neisseria meningitidis* is a facultative pathogen bacterium which is well founded with a number of adhesion molecules to facilitate its colonization in human nasopharynx track. *Neisseria meningitidis* is a major cause of mortality from severe meningococcal disease and septicemia. *Neisseria meningitidis* adhesion, NadA, is a trimeric autotransporter adhesion molecule which is involved in cell adhesion, invasion, and antibody induction. It is identified in approximately 50% of *N. meningitidis* isolates, and is established as a vaccine candidate due to its antigenic effects. In the present study, we exploited bioinformatics tools to better understand and determine the 3D structure of NadA and its functional residues to select B cell epitopes, and provide information for elucidating the biological function and vaccine efficacy of NadA. Therefore, this study provided essential data to close gaps existing in biological areas. The most appropriate model of NadA was designed by SWISS MODEL software and important residues were determined using the subsequent epitope mapping procedures. Locations of important linear and conformational epitopes were determined and conserved residues were identified to broaden our knowledge of efficient vaccine design to reduce meningococcal infectioun in population. These data now provide a theme to design more broadly cross-protective antigens.

*Neisseria meningitidis* is a capsulate gram negative diplococcus which can colonize the upper respiratory tract of humans. It is the major responsible of pyogenic meningitis in children and young adults which can cause their death during a short time despite the advances in drug discovery (1-3). It is reported that the number of patients with meningococcal disease varies from 0.5 to 10 per 100,000 persons each year. Relatively, the patient's number can surge to 15 and 400 per 100,000 during epidemics. 5 to 15% of the patients die from the disease, and 25% of survivors suffer from neurological problems (4-6). Therefore, *N. meningitidis* should be accounted as a disastrous pathogen. It was reported that in special occasions the bacterium is able to move throughout the nasopharynx mucosal layer which can inevitably result in an invasive meningococcal disease including septicemia, and fulminate meningitidis (6, 7). To induce disease, the bacterium needs a broad number of properties leading to colonization as well as invasion to the mucosal barriers, and eventually blood, resulting in septicemia followed by interaction with brain cell microvessels, and blood- brain barrier penetration (8-10). Adhesion-associated properties are due to type IV pili (Tfp), and the non-pilus adhesins which comprise opacity proteins Opa and Opc, and the auto transporter proteins App (adhesion penetration protein), NhhA (Neisseria hia homolog), and NadA (Neisseria adhesin A) (9, 11).

There were numerous attempts to vaccine investigation. The first phases of efficient vaccines in adults but not in children were based on purified polysaccharides of four highly virulent serogroups of *N. meningitidis* (A, C, Y, and W-135). The next efforts were glycoconjugate- based vaccines against C serogroup which were highly successful in disease eradication caused by the mentioned serogroup in infants, children, and adults with 90% confidence. It is of paramount to approach surface exposed proteins in vaccine generation. These vaccines not only induce bactericidal antibodies, but may also eliminate further meningococcal diseases. Mentioned vaccines have been utilized in some countries but there are obstacles which should be tackled. The most important one is their weakness in supporting provision against heterologous strains due to the high sequence variability of protein antigens (12-15).

In this study we focused on NadA, a potential virulence factor, an adhesion molecule, and vaccine candidate. NhhA and NadA are members of the oligomeric coiled-coil adhesion (Oca) family, also named trimeric auto transporters. NadA contributes to bacterial virulence due to its expression in near 50% of disease-associated isolates (8). More than 80% of isolates in hyper virulent lineages have nadA gene, and about half of the meningococcal isolates generate NadA (16, 17). NadA antigen is also reported to be present in 52 out of 53 strains of hypervirulent lineage electerophoretic types (18). Statistical association studies confirm this fact. In disparate geographical locations and time periods, isolates from the same clonal features have similar profiles for this antigen (19). Immunogenic nature of NadA is considerable, while it stimulates the immune system to produce high levels of bacterial antibodies and can be recognized by children's serum antibodies after invasive menangococcal disease (IMD) (20, 21). The gene encoding NadA is associated with strains that belong to three of the four hypervirulent serogroup B (22, 23). Lineages amino acids in C-terminal part have high similarity with that of the Yersinia adhesion YadA with special structure (17, 24). The rate of nadA expression differs among isolates, and can be up to 100-fold. Moreover, it will be over expressed under *in vivo* condition due to the existence of special signals which are associated with the appropriate niche (25, 26). NadA sequences are geneticaly divided into two categories with 45-50% amino acid identity. The first group consists of NadA1, NadA2, and NadA3 which are the most prevalent variants. It is exhibited that their sequence identity rate is 95%, and are cross-reactive from an immunogenic perspective. NadA4, NadA5, and NadA6 are members of the second group which are not common variants (24). In this report, we employed bioinformatics tools for 3D structure investigation regarding the fact that experimental determination is a considerable challenge since it is time-consuming, and its failure rate is relatively high (27). The first step in drug designing is *in silico* study to evaluate capability of candidate molecules. *In silico* studies can be used to determine the target structures for possible binding sites, identify candidate molecules, evaluate their capability to be considered as a drug, dock these molecules with the target, rank them according to their binding affinities, and refine structures to improve binding characteristics (28).

For outer membrane proteins such as NadA, purification and crystallization are cumbersome problems in addition to mentioned obstacles. Taken together, bioinformatics utilization can be more advantageous, and structure investigation is one of the wide applications of these tools in antigen determination and epitope mapping for designing new generation and highly effective vaccines.

## Materials and methods

**Sequence availability, homology search and alignment**

NadA protein sequence with ACM47292.1 accession number containing 362 amino acids was obtained from NCBI at http://www.ncbi.nlm.nih. gov/ protein, and saved in FASTA format for the further analysis. http://blast.ncbi.nlm.nih.gov/Blast. cgi was utilized for mentioned protein BLAST against non-redundant protein database as well as obtaining probable putative conserved domains of the protein. PRALINE (29) at http://www.ibi.vu.nl/ programs/pralinewww/ was used for generation of alignments. Alignments are used to investigate the conservancy of the protein amino acids among various strains. In this regard, suitable vaccine candidates are those effective against all strains of a given pathogen. Conservancy of the protein amino acids among bacteria other than *Neisseria* implies probable cross-reactivity levels.

**Homologous structure search**

To provide template structures the query protein sequence was used as an input data for the PSI-BLAST against protein data bank (PDB) at http://blast.ncbi.nlm.nih.gov/Blast.cgi. 

**General sequence characteristics**

Protein sequence properties including molecular weight, theoretical pI, amino acid composition, total number of negatively and positively charged residues, instability index, and aliphatic index were obtained from an online software at http://expasy. org/tools/protparam. html, Protparam (30).

**Topology prediction**

PREDTMBB server (http://biophysics.biol. uoa.gr/PRED-TMBB/) was recruited for transmembrane 𝛽-strands prediction in protein sequences. The web server could find the topology of the loops in addition to localizing the transmembrane strands. Prediction of hydrophobic transmembrane regions in a protein sequence generates probable 𝛽-barrels that could help determine the 3D protein structure. Topology prediction servers predict the probability of a particular residue to be located either inside or outside of the membrane. Transmembrane helix and signal peptides are not suitable regions as B cell epitope. SPOCTOPUS (31) at http://octopus.cbr. su.se/ was also employed to determine membrane protein topology and signal peptides.

**Secondary structure prediction**

Self-optimized prediction method (SOPM) has been described to improve the efficiency of protein secondary structure prediction. The secondary structure of protein including helix, sheet, turn, and coil parameters was predicted at http://npsa-pbil.ibcp.fr/ cgibin/ npsa automat. pl? page = npsa sopma. html.

**Modeling methods**

Phyre2 (32) at http://www.sbg.bio.ic. ac.uk/ phyre2/ html/ page.cgi? id=index uses hidden markov model alignments through HH search to notably enhance accuracy of alignment and detection rate. To model regions with no detectable homology, phyre2 integrates a new ab initio folding simulation called Poing.

The SWISS- MODEL workspace at http:// swiss-model.expasy.org/ is a web- based incorpo-rated service specialized in protein structure homology modelling.

**Model evaluation**

All 3D models of the protein built, were qualitatively estimated by GMQE and QMEAN4 scores. Qualitative evaluation of 3D models was done by ProSA at https://prosa.services.came. sbg.ac.at (33). ProSA specifically faces the needs confronted in the authentication of protein structures acquired from X-ray analysis, NMR spectroscopy, and hypothetical estimations.

Rampage (34) at http://mordred.bioc.cam.ac. uk/rapper/rampage.php was also employed for estimation of model quality using Ramachandran plot which is an algorithm for atomic level, high-resolution protein structure improvement. It can begin from either C-alpha trace, main-chain model or full-atomic model. ModRefiner (35) at http://zhanglab.ccmb.med. umich.edu/ModRefiner/ is another algorithm with functions similar to Rampage.

**Ligand binding site prediction**

Cofactor at http://zhanglab.ccmb.med.umich. edu/COFACTOR/ is a structure-based method for biological purpose notation of protein molecules. Important amino acids involved in ligand binding site are predicted by this server.

**Functional and structural critical residues identification**

Functional conserved residue allows more realism and robustness in the description of protein binding surfaces and epitope prediction. There are several ways to functionally and structurally annotate/predict important residues.

In this study, InterProSurf at http://curie.utmb. edu/pattest9.html was employed to predict functional sites on protein surface using patch analysis. NadA structure determined by previous strategies, served as an input file for this server. Conseq (36) at http://conseq.tau.ac.il/ utilized NadA sequence as an input. The software parameters were set as follows: PSI-BLAST for five iterations against Uniprot database with E-value of 0.01, and maximum likelihood (ML) as a method of calculating amino acid conservation score.

**Single-scale amino acid properties assay**

Segments within NadA sequence that are likely to be antigenic were predicted using Bcepred at http://www.imtech.res.in/raghava/bcepred with accuracy of 58.7%. This server predicts B-cell epitopes using single of the physico-chemical properties (hydrophilicity, flexibility, mobility, accessibility, polarity, exposed surface and turns) or combination of them. Parameters such as hydrophilicity, flexibility, accessibility, turns and antigenic propensity of polypeptide have been correlated with the location of B cell epitopes. 

**Cleft and cavity analysis**

Profunc at http://www.ebi.ac.uk/thornton-srv/ databases /profunc/ was used to predict clefts and grooves in the protein surface. Depth of clefts and amino acids located within the clefts are predictable by this software.

**Sequence-based B cell epitope prediction**

Two servers were used to ascertain B cell epitopes in NadA. ABCpred at http://www.imtech. res.in/raghava/abcpred/ was used to predict B cell epitope in an antigen sequence.. Window length used for prediction with 0.80 threshold set as 16-mer and overlapping filter was on. BepiPred at http://www.cbs.dtu.dk/services/BepiPred/ was used to predict the location of linear B-cell epitopes.

**Structure-based B cell epitope prediction**

NadA 3D structure served as an input file for servers predicting B cell epitopes based on 3D structure of a given protein. EPCES at http://sysbio.unl.edu/EPCES/ was used to predictp antigenic epitopes on protein surfaces. Ellipro at http://tools.immuneepitope.org/tools/ElliPro/tutorial.jsp was used to predict linear and discontinuous antibody epitopes. 

**Pocket and binding site detection**

GHECOM (Grid-based HECOMi finder) at http://strcomp.protein.osaka-u.ac.jp/ghecom/ was used to find multi-scale pockets on protein surfaces.

## Results

**Sequence availability and alignments**

**Fig. 1:**
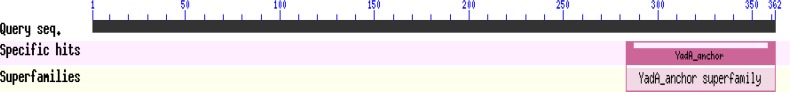
NadA conserved domain. YadA- anchor superfamily is shown

NadA protein sequence with accession number ACM47292.1 and GI:222159579 was obtained, and saved in FASTA format. BLAST search revealed numerous hits to the NadA subunit sequence. Putative conserved domains have been detected. Most of the sequences belonged to YadA super family Protein. Putative conserved domains within this sequence are shown in Figure 1.

**Template search**

The first illustrated result of PSI-BLAST against PDB data bank with 46% identity, 26% query coverage, 77.4 max score and 77.4 total score, chain A, crystal structure of *Neisseria Meningitidis* trimeric autotransporter and vaccine antigen NadA with "4CJD" PDB ID code was opted as template for homology and other modeling strategies. With regard to E-value, max score identification was also influenced by query coverage and max identity. An appropriate hit for template usage is the one with lower E-value, higher query coverage, and max identity. Alignment strategy has been utilized to manifest sequence similarities between the s elected target and other probable templates. Alignment provides a golden opportunity to apply the predicted model to other NadA proteins from other close species.

**Fig. 2:**
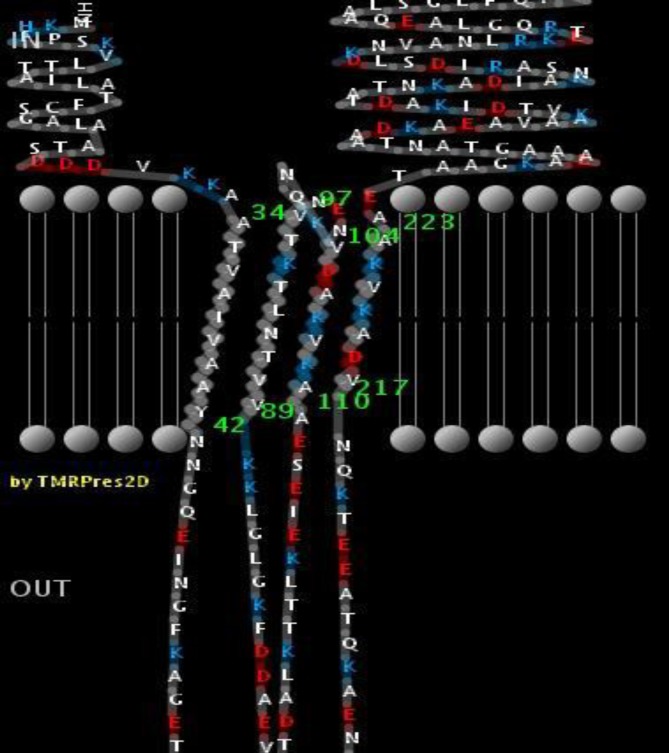
**2D topology model of NadA predicted by TMBBPred server.** Residues from 34-42, 89-97, 104-110 and 217-223 are transmembrane regions

**Fig. 3:**
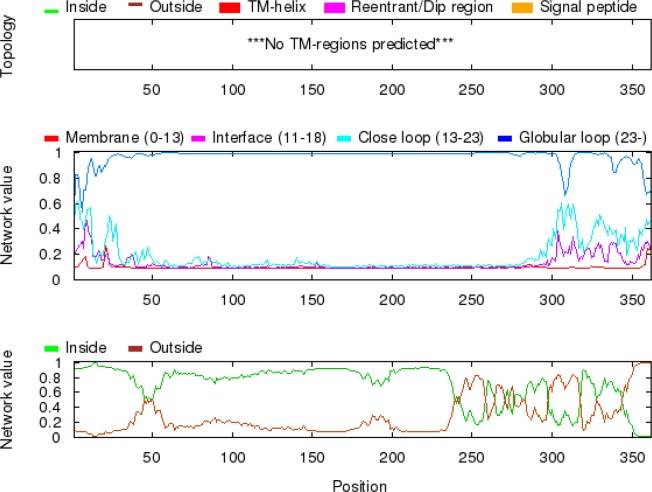
**Graphical representation of the most likely topology predicted by SPOCTOPUS. **Network output: The two diagrams show the estimated preference for each residue to be located in different structural regions. The top diagram shows the preference of being either in (1) the hydrophobic part of the membrane, 0–13 A ° from the membrane center (M), (2) the membrane water-interface, 11–18 A ° from the membrane center (I), (3) a close loop region, 13–23 A ° from the membrane center (L), (4) a globular region, further than 23 A ° from the membrane (G). The bottom diagram shows the estimated preference of a particular residue to be located either on the inside (i) or on the outside (o) of the membrane. The raw data underlying these two plots can be found in the OCTOPUS network file

**Sequence characteristics**

Physical and chemical properties of the selected sequence were predicted. NadA consists of 57 negatively charged residues (Asp+Glu), and 41 positively charged residues (Arg+Lys). Parameters such as molecular weight, theoretical pI (isoelectric point), instability index, aliphatic index, and grand average of hydropathicity which indicate the solubility of the proteins (positive GRAVY (hydrophobic), negative GRAVY (hydro- philic)) are summarized in Table1.

**Secondary structure and topology assessment**

A 2D topology model of NadA was built based on predicted inside, transmembrane, and outside regions of the protein (Fig 2). Coil, helix, and strands are components constituting secondary structure of the protein. The secondary structure can be used to confirm the tertiary structures. Attributions of the secondary structure components in the protein are alpha helix (67.13%), extended strand (11.6%), beta turn (6.35%), and random coil (14.92%). Transmembrane helix does not have antigenic capability and Spoctopus server results did not detect any such helix (Fig.3).

**Table1:** physical and chemical parameters of NadA

**Number of amino acids**	362							
**Molecular weight**	37753.74							
**Theoretical pI**	4.79							
**Amino acid composition**	Ala (A)	71		19.6%/	Arg (R)		5	1.4%
	Asn (N)	24		6.6%/	Asp (D)		29	8.0%
	Cys (C)	1		0.3%/	Gln (Q)		8	2.2%
	Glu (E)	28		7.7%/	Gly (G)		22	6.1%
	His (H)	3		0.8%/	Ile (I)		16	4.4%
	Leu (L)	19		5.2%/	Lys (K)		36	9.9%
	Met (M)	1		0.3%/	Phe (F)		11	3.0%
	Pro (P)	2		0.6%/	Ser (S)		14	3.9%
	Thr (T)	38		10.5%/	Trp (W)		1	0.3%
	Tyr (Y)	6		1.7%/	Val (V)		27	7.5%
	Pyl (O)	0		0.0%/	Sec (U)		0	0.0%
**Total number of negatively charged residues (Asp +Glu)**	57							
**Total number of positively charged residues (Arg +Lys)**	41							
**Atomic composition**	Carbon		C			1634		
	Hydrogen		H			2640		
	Nitrogen		N			452		
	Oxygen		O			567		
	Sulfur		S			2		
**Formula**/**Total number of atoms**	**:** C_1634_H_2640_N_452_O_567_S_2 _/ 5295							
**Extinction coefficients** Extinction coefficients are in units of M^-1^ cm^-1^, at 280 nm measured in water. Ext. coefficient Abs 0.1% (=1 g/l) 0.382, assuming all pairs of Cys residues form cystines Ext. coefficient Abs 0.1% (=1 g/l) 0.382, assuming all Cys residues are reduced	14440 14440							
**Estimated half-life** The N-terminal of the sequence considered is M (Met).	The estimated half-life is: 30 hours (mammalian reticulocytes, in vitro). >20 hours (yeast, in vivo). >10 hours (Escherichia coli, in vivo).							
**Instability index** The instability index (II) is computed to be 12.45	This classifies the protein as stable							
**Aliphatic index**/**Grand average of hydropathicity (GRAVY)**	78.95/ -0.337							

**Fig. 4:**
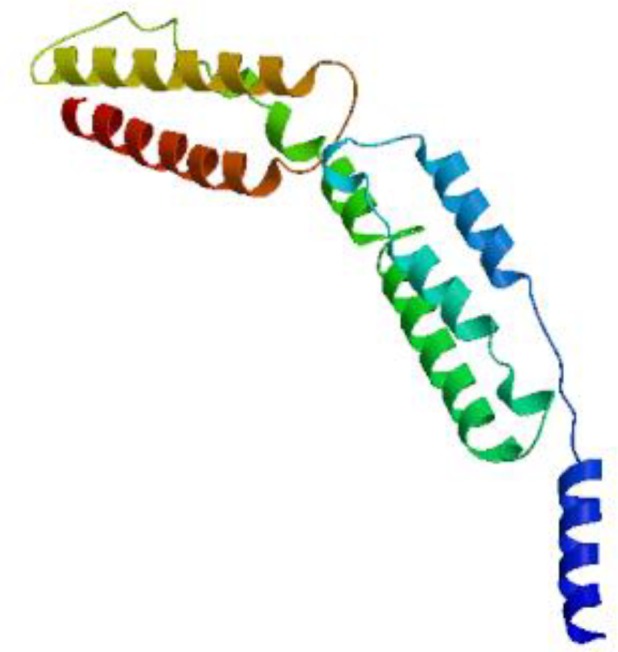
NadA 3D structure model predicted by SWISS MODEL

**Fig. 5:**
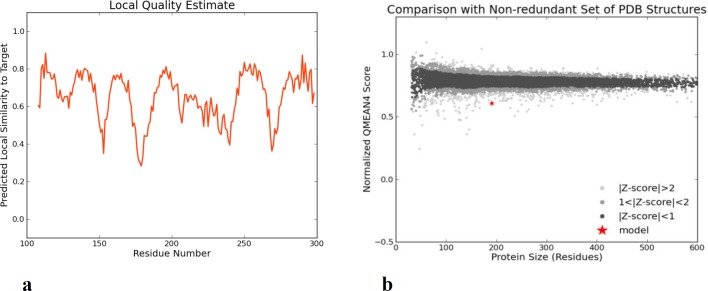
**Model validations.** Both global and local estimation of the quality of the obtained model are reasonable

**Fig. 6:**
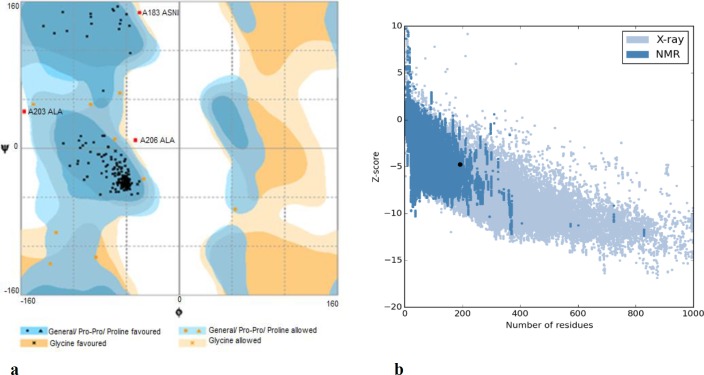
**Model evaluation. **(a) Ramachandran plot of final NadA model. Number of residues in favored region: 177 (93.7%). Number of residues in allowed region: 9 (4.6%). Number of residues in outlier region: 3 (1.8%). (b)- Prosa protein structure anlysis results. Z score = -4.75. Overall quality of the ultimate model is acceptable

**Fig. 7:**
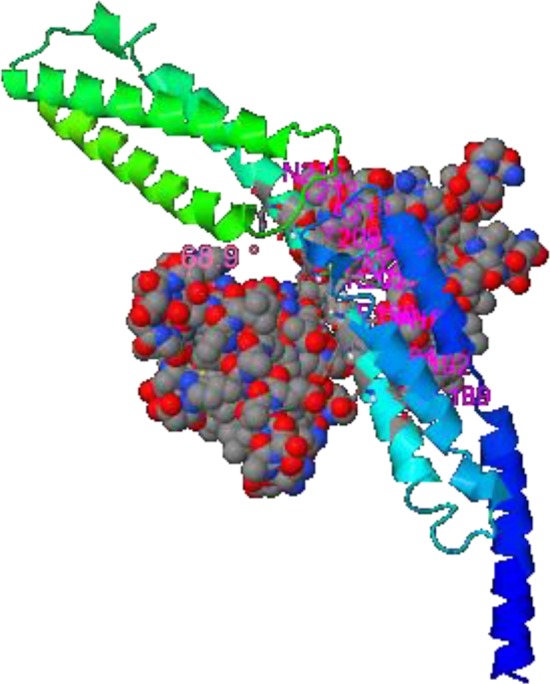
**NadA ligand binding sites predicted by COFACTOR. **NadA structure in contact with peptide ligand from lateral view. Ligand in the space filling model

**Fig. 8:**
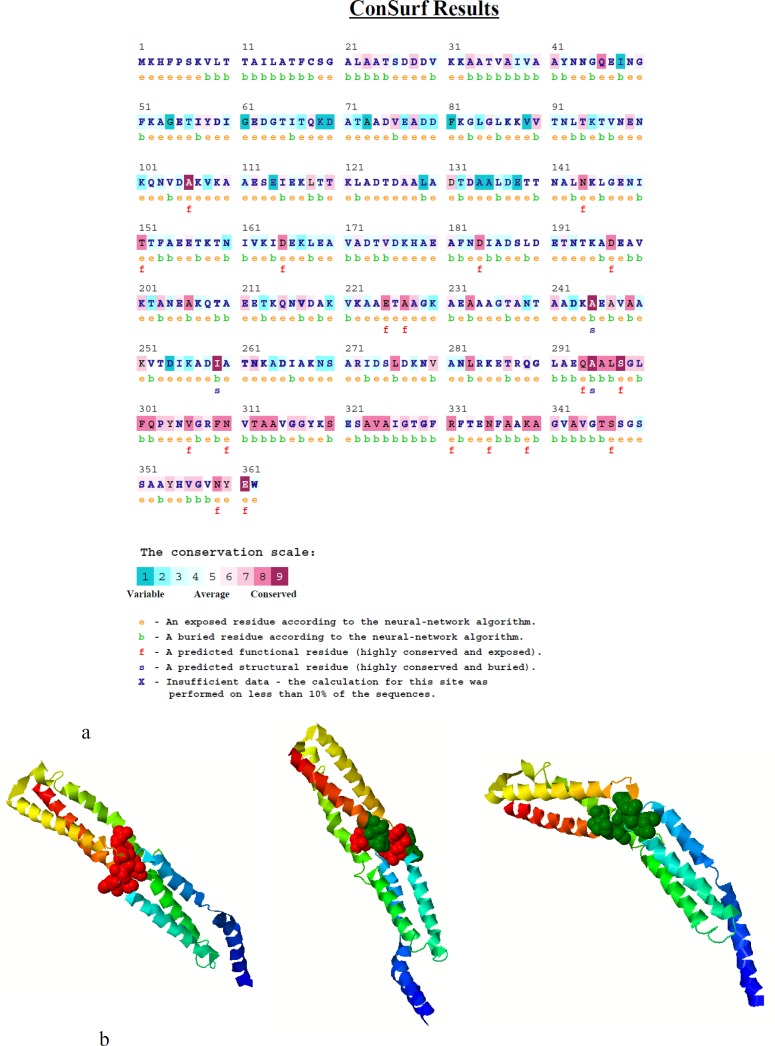
**Functional and structural critical residues. **(a) Colored illustration of conserved functional and structural residues predicted by conSeq server. The evolutionary rate of the sequence is obvious by color scale (see legend). Burial status of the location is labeled by b (buried) and e (exposed). Functionally or structurally importance of the residues were shown by f and s respectively. (b) Different direction of functional residues at the protein structure surface predicted by interproSurf

**Fig. 9:**
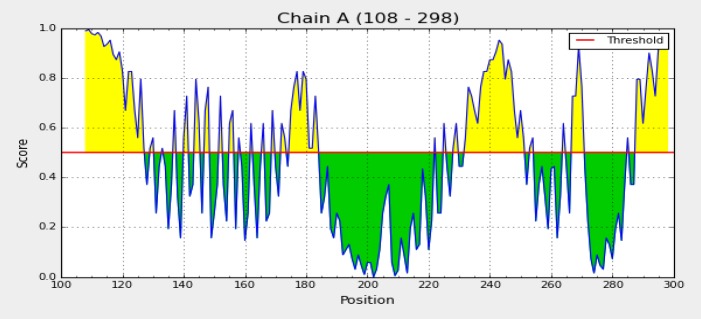
Linear B cell epitopes predicted by Bepipred server

**Fig10:**
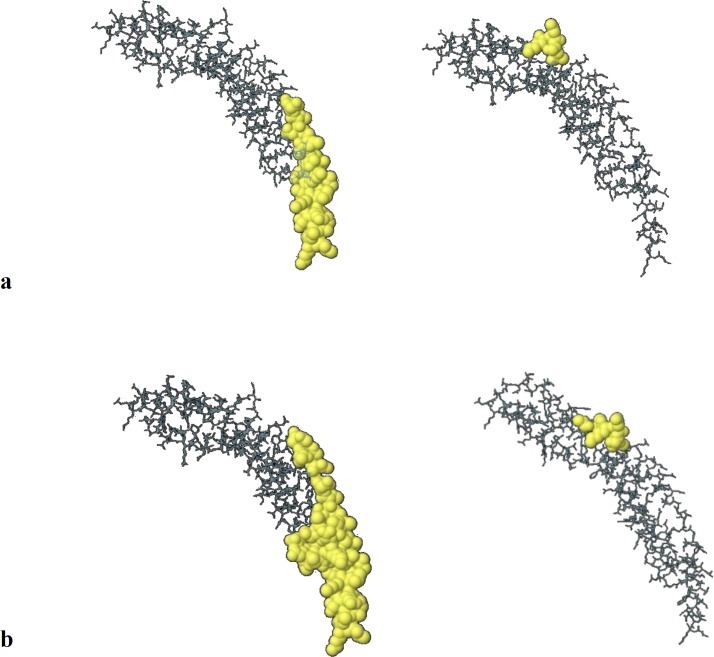
**B cell epitopes** **with the highest PI score.** 2 linear (a) and 2 discontinuous (b) predicted epitopes are represented. Epitopes were mapped on 3D models using Discovery Studio Visualizer 2.5.5 software

**Fig 11:**
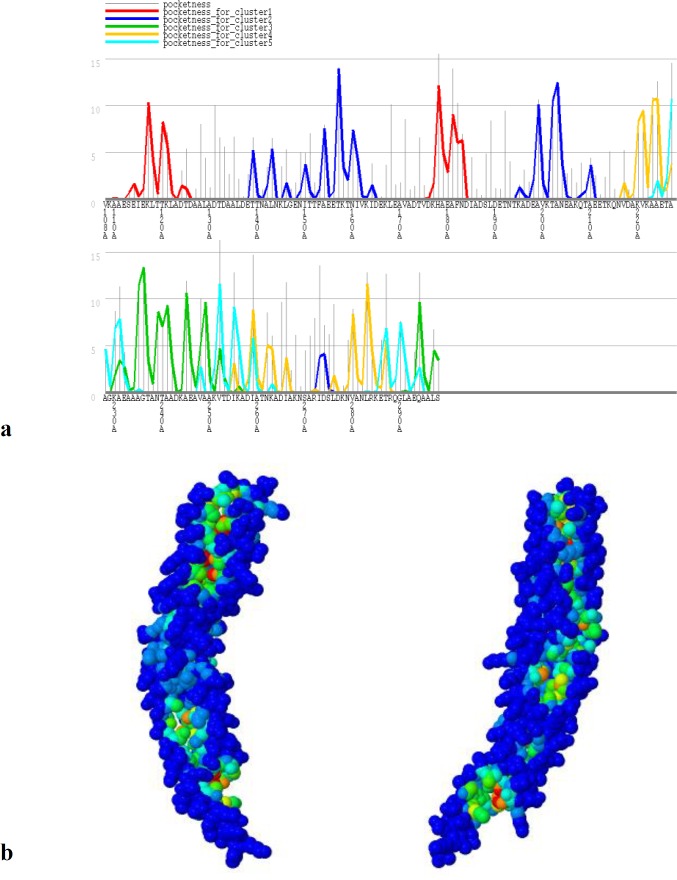
GHECOM results for NadA Pocket detection.

(a) Graph residue-based pocketness. The height of the bar shows the value of pocketness [%] for each residue. The color of pocketness bar indicates cluster number of pocket (red: cluster 1, blue: cluster 2, green: cluster 3, yellow: cluster 4, cyan: cluster 5). (b) Jmol view of pocket structure based on pocketness color

**Homology modeling**

Swiss model and phyre2 software were recruited for homology modeling. The former prepared 3 different models, and the latter introduced one model. All of them were selected for further analyses. The identity score was 46%.

**Model evaluation**

The best 3D model is shown in Figure 4, and model validations are shown in Figure 5. To tackle a number of problems including model structural distortions, plus steric clashes, unphysical phi/psi angles, and irregular hydrogen bonding (H-bonding) networks, all models were evaluated by Rampage and Propsa servers. Regarding the Ramachandran plot, 93.7% of the residues are in favored regions, and 4.8% are in allowed parts while the mere amount of 1.6% are existing in outlier regions (Fig. 6a). Prosa revealed that the predicted model was among other acceptable proteins with z score = -4.75. (Fig. 6b).

**Ligand binding site regions**

Cofactor results illustrated cooperation of conserved residues in ligand binding site (189, 192, 194, 196, 197, 198, 200, 201, 202, 203, 209, 210, 216) with the highest C score LB which indicates the confidence of predicted binding site. Local similarity (sequence & structure) assessment between template binding site and predicted binding site in the query structure showed score 1.02 (BS-score>1) that represents a significant local match between the predicted and template binding site (Fig. 7).

**Functional and structural critical residues**

ConSurf and interproSurf annotated functional residues on the 3D structure of NadA. Results are shown in Figures 8a and b, respectively. ConSurf showed that majority of conserved residues are located in C-terminal region and 17 residues are predicted to be functionally important while residues number 245, 259 and 295 are critical from structure perspective. InterproSurf revealed residues number 150,151, 153,154,213,271,272 272,273, 274,275,276,152,155 by auto patch analysis.

**Table 2:** Bcepred hydrophilicity, accessibility, antigenicity, flexibility and beta turn secondary structure prediction in the protein sequence

Sequence	MKHFPSKVLTTAILATFCSGALAATSDDDVKKAATVAIVAAYNNGQEINGFKAGETIYDIGEDGTITQKDATAADVEADDFKGLGLKKVVTNLTKTVNENKQNVDAKVKAAESEIEKLTTKLADTDAALADTDAALDETTNALNKLGENITTFAEETKTNIVKIDEKLEAVADTVDKHAEAFNDIADSLDETNTKADEAVKTANEAKQTAEETKQNVDAKVKAAETAAGKAEAAAGTANTAADKAEAVAAKVTDIKADIATNKADIAKNSARIDSLDKNVANLRKETRQGLAEQAALSGLFQPYNVGRFNVTAAVGGYKSESAVAIGTGFRFTENFAAKAGVAVGTSSGSSAAYHVGVNYEW^362^
Hydrophilicity	MKHFPSKVLTTAILATFCSGAL**AATSDDDVKKAA**TVAIVA**AYNNGQEIN**GFKAGETIY**DIGEDGTITQKDATAADVEADDFKG**LGLKKVVTNL**TKTVNENKQNVDAK**VKAAESEIEKLTTKLADTDAALADTDAALDETTNALNKLGENITTF**AEETKTN**IVKIDEKLEAV**ADTVDKHA**EAFNDI**ADSLDETNTKADEAVKTANEAKQTAEETKQNVDAK**VKAAETAAGKAEAAAG**TANTAADKAEA**VAAKVTDIKADIATNKADIAKNSARIDSLDKNVANL**RKETRQG**LAEQAALSGLFQPYNVGRFNVTAAV**GGYKSESA**VAIGTGFRFTENFAAKAGVA**VGTSSGSSAA**YHVGVNYEW^362^
Flexibility	MKHFPSKVLTTAILATFCSGALAATSDDDVKKAATVAIVAAYNNGQEINGFKAGETIYDIGEDGTITQKDATAADVEADDFKGLGLKKVVTNLT**KTVNENK**QNVDAKVKAAESEIEKLTTKLADTDAALADTDAALDETTNALNKLGENIT**TFAEETK**TNIVKIDEKLEAVADTVDKHAEAFNDIA**DSLDETN**TKADEAVKTANEAK**QTAEETK**QNVDAKVKAAETAAGKAEAAAGTANTAADKAEAVAAKVTDIKADIATNKADIAKNSARIDSLDKN**VANLRKETR**QGLAEQAALSGLFQPYNVGRFNVTA**AVGGYKS**ESAVAIGTGFRFTENFAAKA**GVAVGTSSGS**SAAYHVGVNYEW^362^
Accessibility	**MKHFPSKV**LTTAILATFCSGALAA**TSDDDVKKAA**TVAIVA**AYNNGQEIN**GFKAGETIYDIGE**DGTITQKDATA**ADVEADDFKGLGLKKVVTN**LTKTVNENKQNVDAK**VKA**AESEIEKLTTKLADTD**AALADTDAALDE**TTNALNK**LGENIT**TFAEETKTNIVKIDEKLE**AVAD**TVDKHAE**AFNDIA**DSLDETNTKADEAVKTANEAKQTAEETKQNVDAKVKAAET**AAGKAEAAAGTANTAADKAEAVAAKVTDIKADIAT**NKADIAKNSARIDSLDKNVANLRKETRQGLAEQ**AALSGLF**QPYNVGR**FNVTAAV**GGYKSESA**VAIGTGFRFTENFAAKAGVAVGTSSGSSAAYHVGVNYEW^362^
Turns	MKHFPSKVLTTAILATFCSGALAATSDDDVKKAATVAIVAAYNNGQEINGFKAGETIYDIGEDGTITQKDATAADVEADDFKGLGLKKVVTNLTKTVNENKQNVDAKVKAAESEIEKLTTKLADTDAALADTDAALDETTNALNKLGENITTFAEETKTNIVKIDEKLEAVADTVDKHAEAFNDIADSLDETNTKADEAVKTANEAKQTAEETKQNVDAKVKAAETAAGKAEAAAGTANTAADKAEAVAAKVTDIKADIATNKADIAKNSARIDSLDKNVANLRKETRQGLAEQAALSGLFQPYNVGRFNVTAAVGGYKSESAVAIGTGFRFTENFAAKAGVAVGTSSGSSAAYHVGVNYEW^362^
Exposed Surface	MKHFPSKVLTTAILATFCSGALAAT**SDDDVKKA**ATVAIVAAYNNGQEINGFKAGETIYDIGEDGTITQKDATAADVEADDFKGLGLKKVVTNLT**KTVNENKQNVDAK**VKAAESEIEKLTTKLADTDAALADTDAALDETTNALNKLGENITTFAEETKTNIV**KIDEKL**EAVADTVDKHAEAFNDIADSLDETNTKADEAV**KTANEAK**QT**AEETKQNVDAK**VKAAETAAGKAEAAAGTANTAADKAEAVAAKVTDIKADIATNKADIAKNSARIDSLDKNVA**NLRKETRQG**LAEQAALSGLFQPYNVGRFNVTAAVGGYKSESAVAIGTGFRFTENFAAKAGVAVGTSSGSSAAYHVGVNYEW^362^
Polarity	**MKHFPSKV**LTTAILATFCSGALAA**TSDDDVKKAA**TVAIVAAYNNGQEINGFKAGETIYDIGEDGTITQKDATA**ADVEAD**DFKGLGLKKVVTNLT**KTVNENK**QNVDAKVKA**AESEIEKLTTKL**ADTDAALADTDAALDETTNALNKLGENIT**TFAEE**TKTNIVKIDEKLEAVADTVDKHAEAFNDIADSLDETNT**KADEAVKTANEAKQTAEETKQNV**DAKVKAAETAAGKAEAAAGTANTAADKAEAVAAKVTDIKADIATNKADIAKNSA**RIDSLDK**N**VANLRKETRQGL**AEQAALSGLFQPYNVGRFNVTAAVGGYKSESAVAIGTGFRFTENFAAKAGVAVGTSSGSSAAYHVGVNYEW^362^
Antigenic Propensity	MK**HFPSKVLT**TAILATFCSGALAATSDDDVKKAATVAIVAAYNNGQEINGFKAGETIYDIGEDGTITQKDATAADVEADDFKG**LGLKKVVT**NLTKTVNENKQNVDAKVKAAESEIEKLTTKLADTDAALADTDAALDETTNALNKLGENITTFAEETKTNIVKIDEKLEAVADTVDKHAEAFNDIADSLDETNTKADEAVKTANEAKQTAEETKQNVDAKVKAAETAAGKAEAAAGTANTAADKAEAVAAKVTDIKADIATNKADIAKNSARIDSLDKNVANLRKETRQGLAEQAALSG**LFQPYNV**GRFNVTAAVGGYKSESAVAIGTGFRFTENFAAKAGVAVGTSSGSSAA**YHVGVNY**EW^362^

Bold sequences indicate mentioned characteristics in protein sequence.

**S** **ingle-scale amino acid properties assay**

Properties such as hydrophilicity, accessi-bility, antigenicity, flexibility, and beta turn secondary structure in the protein sequence were predicted by Bcepred server (Table 2). Single-scale amino acid properties were detectable in all sequence length. Query sequence is shown from number 1, and each property has been shown with a distinct color. According to the mentioned characteristics, middle regions of the protein are more likely to have B cell epitope properties. "TSDDDVKKAA", "AEETKTN", "KADEAVKT-ANEAKQTAEETKQNV" residues are capable to be B cell epitopes.

**Cleft analysis**

ProFunc results showed a location with 12.17A depth, 57.64% accessible vertices, and 13.66% buried vertices that are the major cleft in NadA.

**Prediction of B cell epitopes by integrated strategy**

B-cell epitopes are regions on the surface of antigens that are recognized by B-cell receptors or specific antibodies. These epitopes can be categorized into two types: linear (continuous) and conformational (discontinuous) epitopes. Linear B cell epitopes were predicted by Bepipred server (Fig. 9). ABCpred result showed 9 hits of 16 mer peptide sequences as B-cell epitopes with ranking based on scores (Table 3).

The predicted B cell epitopes were ranked according to their score obtained by trained recurrent neural network, with higher score of the peptide corresponding to the higher probability to be an epitope. All shown peptides were above the threshold value chosen (0.80). Linear and discontinuous B cell epitopes were predicted by ElliPro software (Tables 4 and 5). Two discontin-uous and 2 linear epitopes with the highest PI (protrusion index) are shown in Figures 10a and 10b.

**Table 3:** Predicted linear epitopes (Linear B cell epitope predicted by ABCpred server).

**Rank**	**Sequence**	**Start position**	**Score**
1	TITQKDATAADVEADD	65	0.95
2	GETIYDIGEDGTITQK	54	0.94
3	DETNTKADEAVKTANE	190	0.92
4	IVKIDEKLEAVADTVD	161	0.90
5	TVAIVAAYNNGQEING	35	0.87
6	SGLFQPYNVGRFNVTA	298	0.87
7	AVAIGTGFRFTENFAA	323	0.86
8	YNNGQEINGFKAGETI	42	0.85
9	TTAILATFCSGALAAT	10	0.84
10	DAKVKAAESEIEKLTT	105	0.80

**Table 4:** Linear Epitopes Predicted by Ellipro

**No.**	**Start**	**End**	**Peptide**	**Number of residues**	**Score**
1	108	134	VKAAESEIEKLTTKLADTDAALADTDA	27	0.748
2	267	271	AKNSA	5	0.718
3	172	184	ADTVDKHAEAFND	13	0.651
4	221	254	VKAAETAAGKAEAAAGTANTAADKAEAVAAKVTD	34	0.630
5	155	158	EETK	4	0.51

**Table 5:** Discontinuous Epitopes Predicted by Ellipro

**No.**	**Residues**	**Number of residues**	**Score**
1	A:V108, A:K109, A:A110, A:A111, A:E112, A:S113, A:E114, A:I115, A:E116, A:K117, A:L118, A:T119, A:T120, A:K121, A:L122, A:A123, A:D124, A:T125, A:D126, A:A127, A:L129, A:A130, A:D131, A:T132, A:D133, A:A134, A:D137, A:T140, A:N141, A:N144, A:A172, A:D173, A:T174, A:V175, A:D176, A:K177, A:H178, A:A179, A:E180, A:A181, A:F182, A:N183, A:D184	43	0.722
2	A:A264, A:A267, A:K268, A:N269, A:S270, A:A271	6	0.702
3	A:A219, A:V221, A:K222, A:A223, A:A224, A:E225, A:T226, A:A227, A:A228, A:G229, A:K230, A:A231, A:E232, A:A233, A:A234, A:A235, A:G236, A:T237, A:A238, A:N239, A:T240, A:A241, A:A242, A:D243, A:K244, A:A245, A:E246, A:A247, A:V248, A:A249, A:A250, A:K251, A:T253, A:D254, A:Q289, A:G290, A:L291, A:A292, A:E293, A:Q294, A:A295, A:A296, A:L297, A:S298	44	0.676
4	A:G147, A:E148, A:I150	3	0.564

"VKAAESEIEKLTTKLADTDAALADTDA" at position 108- 134 and "AKNSA" at position 267- 271 were the best linear epitopes determined by Ellipro.

**NadA pocket detection**

GHECOM server found 5 pockets on protein surfaces using mathematical morphology. A residue in a deeper and larger pocket has a greater chance to be a true pocket. The pockets of small-molecule binding sites and active sites were higher than the average value; specifically, the values for the active sites were much higher. This suggests that pockets contribute to the formation of binding sites and active sites of protein. GHECOM results are shown in Figures 11a and b.

## Discussion

There are many advantages for NadA to be an acceptable vaccine candidate. Relatively, NadA is involved in bacterial adhesion due to the surface exposure, and is expressed in 50% of virulent strains and all three hypervirulent lineages. It also induces proinflammatory cytokines (9, 11). NadA based vaccines may plummet the meningococcal disease prevalence. Functional blockade of protein may result in bacterial death. It is believed that antibody utilization to target the functional residues results in disruption of bacterial adhesion to the host epithelial cells (12-15). NadA mediated bacterium invasion and adhesion provided us the rational to determine appropriate regions as a vaccine candidates. Bioinformatics tools were exploited to achieve such goal. Additionally, similarity results revealed that NadA sequences were evolutionary conserved in most of the hypervirulent species suggesting that vaccines based on this protein can be effective against other pathogenic serotypes as well as some other Gram- negative bacteria due to the cross- reactivity of their antibodies (19). According to the NadA location and its chemical characteristics (Table 1), it is an outer membrane and acidic protein (PI= 4.79) which are considered as desirable conditions for B cell response induction. Topology predictions revealed that there is not any transmembrane helix in its structure while it is believed that hidden residues such as transmembrane helixes are not appropriate B cell epitope candidates.

Building a homology model comprises four main steps: identification of the template, alignment, model building, and quality evaluation (37). These steps has been repeated until a satisfactory model was achieved using SWISS MODEL.

The 3D models estimated qualitatively by two servers revealed that there was a consensus on a single model. QMEAN is a composite scoring function for the estimation of the global and local model quality. The score of a model is also shown in relation to a set of high-resolution PDB structures (Z-score).

Altogether, various methods were employed to provide data on conserved residue detection which are involved in ligand binding and protein- protein interactions to be used for further investigations such as site directed mutations to improve the knowledge of vaccine and antibody production. In this study, we combined all the data obtained from various servers and software to predict B-cell epitopes. This study provides information on biophysical structure of NadA which was performed by crystallography in previous studies. These data shed light on 3D structure of this critical antigen. The structure presented here reveals functional models that can improve the interpretation of previous studies and facilitate further researches to elucidate thoroughgoing NadA as both an adhesin and a vaccine antigen. These findings now provide a theme to design more broadly cross-protective antigens.

## Conflict of interest

Authors declared no conflict of interest.

## References

[1] Rouphael NG, Stephens DS (2012). Neisseria meningitidis: biology, microbiology, and epidemiology. Methods Mol Biol.

[2] Yazdankhah SP, Caugant DA (2004). Neisseria meningitidis: an overview of the carriage state. J Med Microbiol.

[3] Khater WS, Elabd SH (2016). Identification of Common Bacterial Pathogens Causing Meningitis in Culture-Negative Cerebrospinal Fluid Samples Using Real-Time Polymerase Chain Reaction. Int J Microbiol.

[4] Gotschlich EC, Liu TY, Artenstein MS (1969). Human immunity to the meningococcus. 3. Preparation and immunochemical properties of the group A, group B, and group C meningococcal polysaccharides. J Exp Med.

[5] Jafri RZ, Ali A, Messonnier NE (2013). Global epidemiology of invasive meningococcal disease. Popul Health Metr.

[6] Trotter C, Ramsay M, Harrison L, Feavers I, Pollard AJ, Sadarangani M (2016). Introduction and epidemiology of meningococcal disease. Handbook of Meningococcal Disease Management.

[7] Slanina H, Hebling S, Hauck CR (2012). Cell invasion by Neisseria meningitidis requires a functional interplay between the focal adhesion kinase, Src and cortactin. PLoS One.

[8] Hill DJ, Griffiths NJ, Borodina E (2010). Cellular and molecular biology of Neisseria meningitidis colonization and invasive disease. Clin Sci (Lond).

[9] Pizza M, Rappuoli R (2015). Neisseria meningitidis: pathogenesis and immunity. Curr Opin Microbiol.

[10] Schoen C, Kischkies L, Elias J (2014). Metabolism and virulence in Neisseria meningitidis. Front Cell Infect Microbiol.

[11] Coureuil M, Lecuyer H, Bourdoulous S (2017). A journey into the brain: insight into how bacterial pathogens cross blood-brain barriers. Nat Rev Microbiol.

[12] Frasch CE, Bash MC, Ellis RW, Brodeur BR (2003). Neisseria meningitidis vaccines. New Bacterial Vaccines.

[13] Pinto VB, Burden R, Wagner A (2013). The development of an experimental multiple serogroups vaccine for Neisseria meningitidis. PLoS One.

[14] Ali A, Jafri RZ, Messonnier N (2014). Global practices of meningococcal vaccine use and impact on invasive disease. Pathog Glob Health.

[15] Gasparini R, Panatto D, Bragazzi NL (2015). How the Knowledge of Interactions between Meningococcus and the Human Immune System Has Been Used to Prepare Effective Neisseria meningitidis Vaccines. J Immunol Res.

[16] Cotter SE, Surana NK, St Geme JW, 3rd (2005). Trimeric autotransporters: a distinct subfamily of autotransporter proteins. Trends Microbiol.

[17] Linke D, Riess T, Autenrieth IB (2006). Trimeric autotransporter adhesins: variable structure, common function. Trends Microbiol.

[18] Comanducci M, Bambini S, Brunelli B (2002). NadA, a novel vaccine candidate of Neisseria meningitidis. J Exp Med.

[19] Bambini S, Muzzi A, Olcen P (2009). Distribution and genetic variability of three vaccine components in a panel of strains representative of the diversity of serogroup B meningococcus. Vaccine.

[20] Findlow J, Borrow R, Snape MD (2010). Multicenter, open-label, randomized phase II controlled trial of an investigational recombinant Meningococcal serogroup B vaccine with and without outer membrane vesicles, administered in infancy. Clin Infect Dis.

[21] Litt DJ, Savino S, Beddek A (2004). Putative vaccine antigens from Neisseria meningitidis recognized by serum antibodies of young children convalescing after meningococcal disease. J Infect Dis.

[22] Vogel U, Taha MK, Vazquez JA (2013). Predicted strain coverage of a meningococcal multicomponent vaccine (4CMenB) in Europe: a qualitative and quantitative assessment. Lancet Infect Dis.

[23] Wang X, Cohn A, Comanducci M (2011). Prevalence and genetic diversity of candidate vaccine antigens among invasive Neisseria meningitidis isolates in the United States. Vaccine.

[24] Comanducci M, Bambini S, Caugant DA (2004). NadA diversity and carriage in Neisseria meningitidis. Infect Immun.

[25] Schielke S, Huebner C, Spatz C (2009). Expression of the meningococcal adhesin NadA is controlled by a transcriptional regulator of the MarR family. Mol Microbiol.

[26] Fagnocchi L, Biolchi A, Ferlicca F (2013). Transcriptional regulation of the nadA gene in Neisseria meningitidis impacts the prediction of coverage of a multicomponent meningococcal serogroup B vaccine. Infect Immun.

[27] Petrey D, Honig B (2005). Protein structure prediction: inroads to biology. Mol Cell.

[28] Saeidnia S, Manayi A, Abdollahi M (2013). The pros and cons of the in-silico pharmaco-toxicology in drug discovery and development. Int J Pharm.

[29] Simossis VA, Heringa J (2005). PRALINE: a multiple sequence alignment toolbox that integrates homology-extended and secondary structure information. Nucleic Acids Res.

[30] Gasteiger E, Hoogland C, Gattiker A, Walker JM (2005). Protein Identification and Analysis Tools on the ExPASy Server. The Proteomics Protocols Handbook.

[31] Viklund H, Bernsel A, Skwark M (2008). SPOCTOPUS: a combined predictor of signal peptides and membrane protein topology. Bioinformatics.

[32] Kelley LA, Sternberg MJ (2009). Protein structure prediction on the Web: a case study using the Phyre server. Nat Protoc.

[33] Wiederstein M, Sippl MJ (2007). ProSA-web: interactive web service for the recognition of errors in three-dimensional structures of proteins. Nucleic Acids Res.

[34] Lovell SC, Davis IW, Arendall WB 3rd (2003). Structure validation by Calpha geometry: phi,psi and Cbeta deviation. Proteins.

[35] Xu D, Zhang Y (2011). Improving the physical realism and structural accuracy of protein models by a two-step atomic-level energy minimization. Biophys J.

[36] Berezin C, Glaser F, Rosenberg J (2017). ConSeq: the identification of functionally and structurally important residues in protein sequences. Bioinformatics.

[37] Saxena A, Sangwan RS, Mishra S (2013). Fundamentals of Homology Modeling Steps and Comparison among Important Bioinformatics Tools. An Overview Sci Int.

